# Severe thrombocytopenia induced by niraparib in ovarian cancer patients: a case report and literature review

**DOI:** 10.3389/fonc.2025.1542646

**Published:** 2025-07-04

**Authors:** Jiajun Li, Jiaxi Yang, Ying Wang

**Affiliations:** ^1^ Department of Pharmacology, Hebei Medical University, Shijiazhuang, China; ^2^ Department of Gynecology, The Fourth Hospital of Hebei Medical University, Shijiazhuang, China

**Keywords:** poly (ADP-ribose) polymerase inhibitor, niraparib, ovarian cancer, thrombocytopenia, case report

## Abstract

Ovarian cancer (OC) is a common gynecological malignancy, and poly (ADP-ribose) polymerase inhibitor (PARPi) maintenance therapy has significantly prolonged the survival of newly diagnosed or platinum-sensitive recurrent ovarian cancer patients, which has become a new treatment paradigm for ovarian cancer. Niraparib is a widely used PARP inhibitor in the clinical setting, and its adverse effects are also a major concern. The common adverse reactions of Niraparib mainly include various hematological adverse reactions (such as thrombocytopenia and anemia), gastrointestinal adverse reactions (such as nausea and vomiting), and fatigue. In previous related reports, thrombocytopenia has been mentioned multiple times, a relatively common adverse reaction of Niraparib. However, there have been no reports of irreversible and persistent thrombocytopenia. This case report describes a 59-year-old woman who developed persistent thrombocytopenia 11 months after adjuvant chemotherapy for stage IIIC high-grade serous ovarian cancer. Despite various treatment measures, the patient’s platelet count continues to fluctuate and remains low. This is a severe thrombocytopenia that may rare adverse reaction caused by Niraparib. This article adds important information to the scientific literature on potential serious adverse reactions in long-term treatment with Niraparib. It emphasizes the necessity of closely monitoring platelet counts during Niraparib treatment.

## 1 Introduction

Ovarian cancer (OC) is one of the most common malignant tumors of the female reproductive tract. According to the latest global cancer burden data in 2024, there are 324,398 new cases and 206,839 deaths from ovarian cancer worldwide each year (1). In China, there are approximately 61,100 new cases and 33,600 deaths from ovarian cancer annually (2). Ovarian cancer can be classified based on the tissue of origin, including epithelial cancers, germ cell malignancies, and sex cord-stromal tumors. Epithelial ovarian cancer is the most common type, accounting for around 90% of ovarian malignancies (3). Due to the deep pelvic location of the ovaries, ovarian cancer often lacks specific early symptoms, leading to late-stage diagnosis in many patients. As a result, the prognosis is generally poor, posing a serious threat to patient survival (4).

Recent advances in ovarian cancer maintenance therapy have shifted the focus toward dual optimization of treatment efficacy and toxicity profiles (5). The expanding knowledge of tumor molecular genetics and clinical implementation of targeted therapies have significantly broadened precision treatment options (6). Contemporary maintenance regimens now strategically combine targeted agents and immunotherapies with conventional approaches, demonstrating superior survival benefits alongside improved safety and quality of life outcomes.

Multiple studies have demonstrated that poly ADP-ribose polymerase (PARP) inhibitors exert a synthetic lethal effect on tumor cells harboring mutations in breast cancer-related genes (BRCA), bringing a new direction to the precision treatment of ovarian cancer (OC) (7). In OC maintenance therapy, PARP inhibitors have been shown to significantly prolong the survival of newly diagnosed or platinum-sensitive recurrent ovarian cancer patients, and have become a new treatment paradigm for ovarian cancer.

First-line maintenance therapy has become an integral component of comprehensive treatment for newly diagnosed ovarian cancer patients, initiated after completion of primary chemotherapy when patients achieve either complete response (CR) or partial response (PR) (8). This therapeutic strategy primarily aims to prolong progression-free survival and improve long-term clinical outcomes by delaying disease recurrence. Regarding treatment options, conventional chemotherapeutic agents have demonstrated limited clinical value in maintenance settings (6). Accumulating evidence from multiple studies has failed to confirm their efficacy, leading to their exclusion from current clinical guidelines. While the anti-angiogenic agent bevacizumab remains a maintenance option, its administration requires concurrent use with primary chemotherapy. Notably, this regimen shows modest clinical benefit in the general patient population, extending progression-free survival (PFS) by only 3–4 months, except in high-risk subgroups with greater propensity for recurrence (5).

Recent years have witnessed groundbreaking advances in first-line maintenance therapy with the publication of multiple phase III randomized controlled trials evaluating PARP inhibitors (9). These studies have consistently demonstrated significant improvements in PFS, with certain molecular subgroups also exhibiting trends toward overall survival (OS) benefit. Based on this robust evidence, PARP inhibitors have been endorsed by major international guidelines as the standard-of-care maintenance therapy for selected molecular subtypes of ovarian cancer (10).

The pharmacological properties of different PARP inhibitors vary. The approved PARP inhibitors each have their advantages in terms of bioavailability, half-life, metabolic enzymes, and tissue distribution (11). Niraparib is a poly (ADP-ribose) polymerase (PARP) inhibitor that primarily acts on PARP-1 and PARP-2 (12). As the first PARP inhibitor approved by the US Food and Drug Administration (FDA) that does not require BRCA gene mutations or other biomarkers, niraparib can effectively inhibit the activity of enzymes related to DNA damage repair. This inhibitory effect hampers the DNA repair process, rendering tumor cells unable to effectively repair their DNA damage, and thereby inducing tumor cell apoptosis and delaying or even halting tumor progression.

The clinical trials of PARP inhibitors in ovarian cancer have yielded several key findings: The SOLO-1 trial showed that olaparib significantly extended progression-free survival (PFS) and overall survival (OS) in patients with BRCA mutations, with results consistent across Chinese and global populations (10). The PAOLA-1 study demonstrated that the combination of olaparib and bevacizumab provided significant PFS and OS benefits for patients with BRCA mutations or HRD-positive disease, but no benefits were observed in HRD-negative patients (13). The PRIMA trial indicated that niraparib significantly improved PFS in patients with BRCA mutations or HRD-positive disease, although no differences in OS were noted. HRD-negative patients experienced limited benefits (12). Lastly, the PRIME trial (conducted in China) revealed significant PFS extension across the entire patient population, including those without residual disease, with individualized dosing showing superior safety (14).

As the clinical application of PARP inhibitors has become increasingly widespread, the associated adverse effects have also drawn considerable attention (15). The adverse events associated with PARP inhibitors include hematological toxicities, gastrointestinal toxicities, and neurological toxicities, with each drug having its unique toxicity profile (16). Of particular concern is thrombocytopenia, as PARP1 is expressed in the megakaryocyte lineage, and PARP inhibition can reduce platelet formation. The clinical manifestations of thrombocytopenia can range from mild mucocutaneous bleeding to severe internal organ hemorrhage, potentially even life-threatening intracranial hemorrhage. The onset of thrombocytopenia can be insidious and easily overlooked.

Notably, we report a case of an ovarian cancer patient who developed persistent, severe thrombocytopenia during maintenance therapy with the PARP inhibitor Niraparib. This case highlights the importance of closely monitoring platelet-related adverse events when using PARP inhibitors, and implementing appropriate surveillance and management strategies.

## 2 Case presentation

A 59-year-old female patient was admitted on May 5, 2023, due to thrombocytopenia discovered 24 days after 11 months of postoperative chemotherapy for stage IIIC high-grade serous ovarian cancer.

### 2.1 Medical history headings

**Figure 1:**
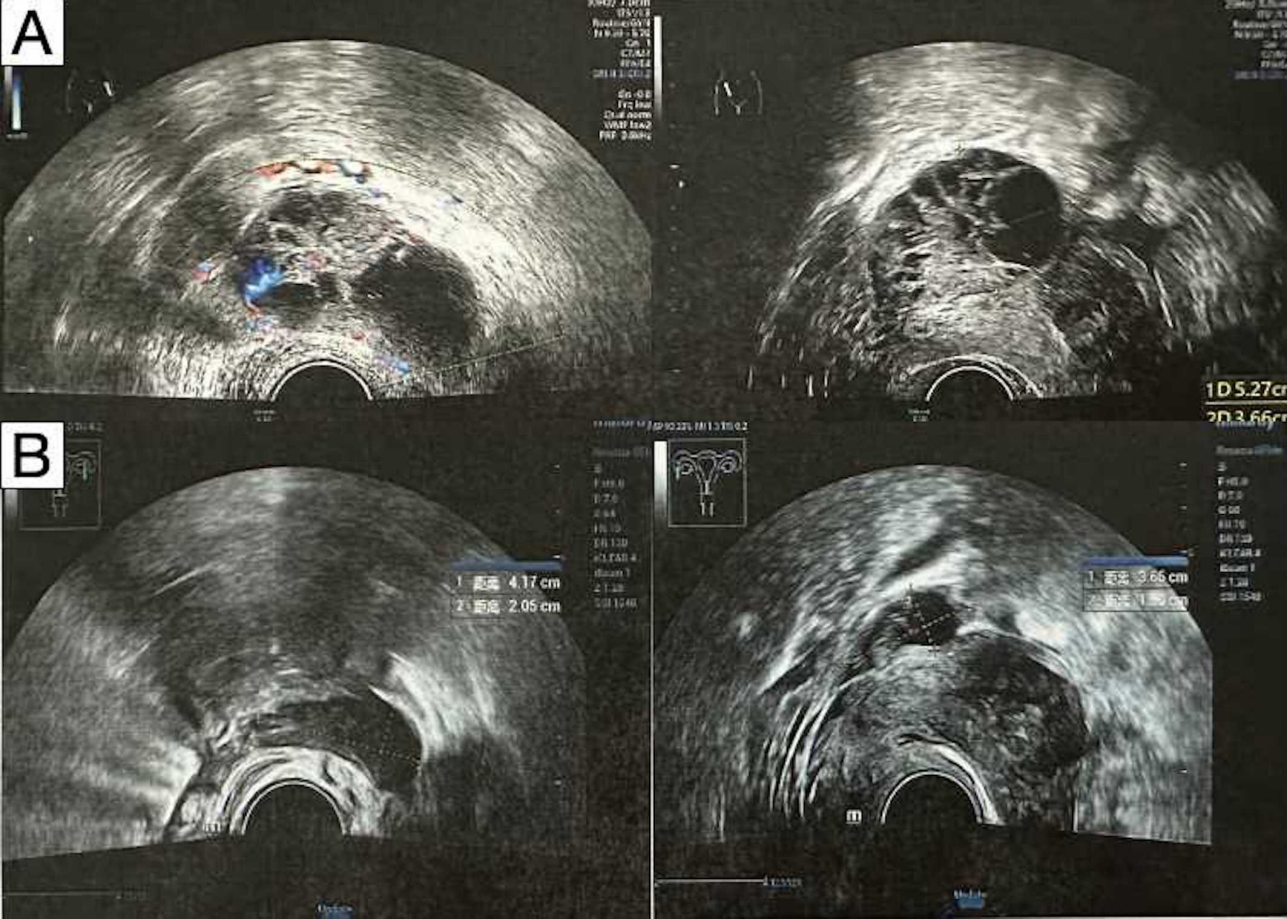
Transabdominal ultrasonography demonstrated bilateral adnexal masses. **(A)** Before neoadjuvant therapy. **(B)** After neoadjuvant therapy.

**Figure 2:**
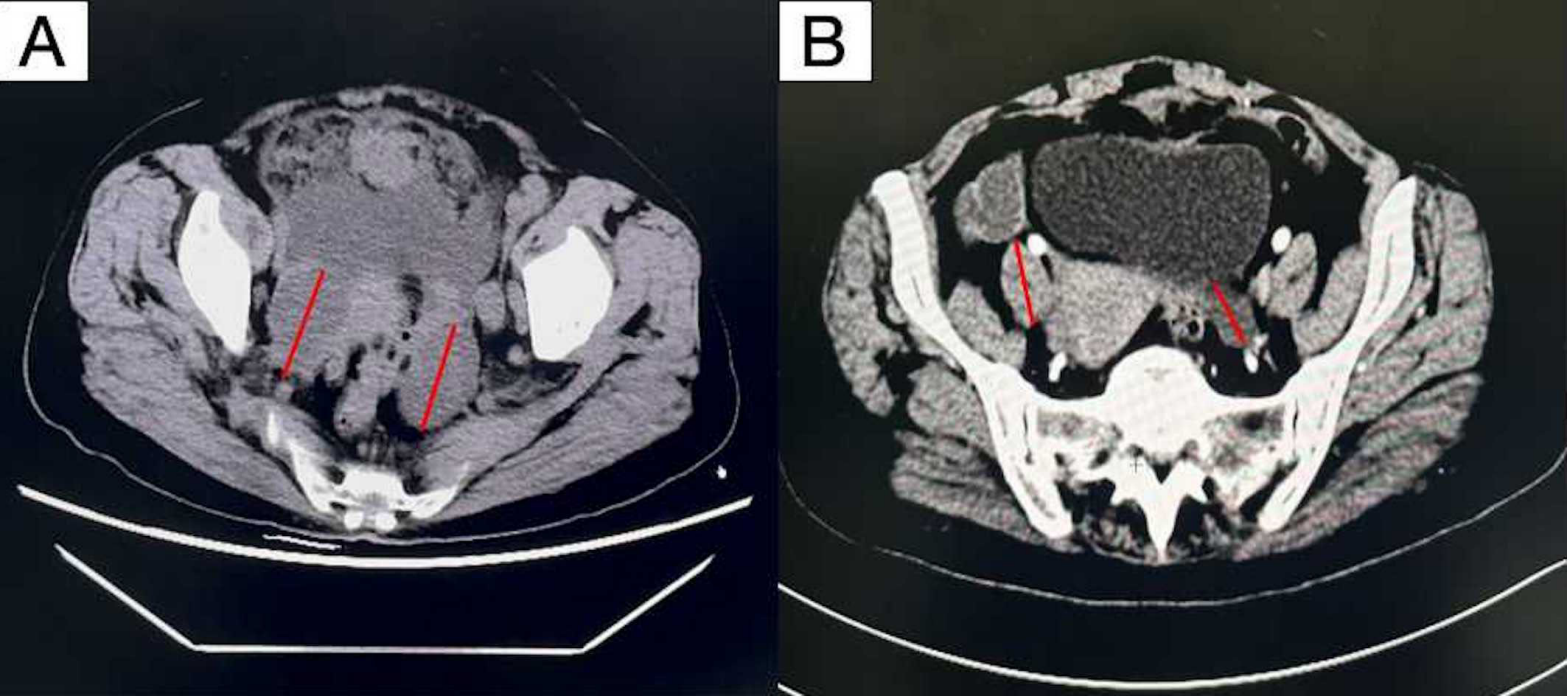
Computed tomography scan showing irregular masses in the bilateral pelvic adnexa area. **(A)** Before neoadjuvant therapy **(B)** after neoadjuvant therapy. By comparing **(A, B)**, it can be seen that the tumor bed significantly shrinks after neoadjuvant chemotherapy.

**Figure 3:**
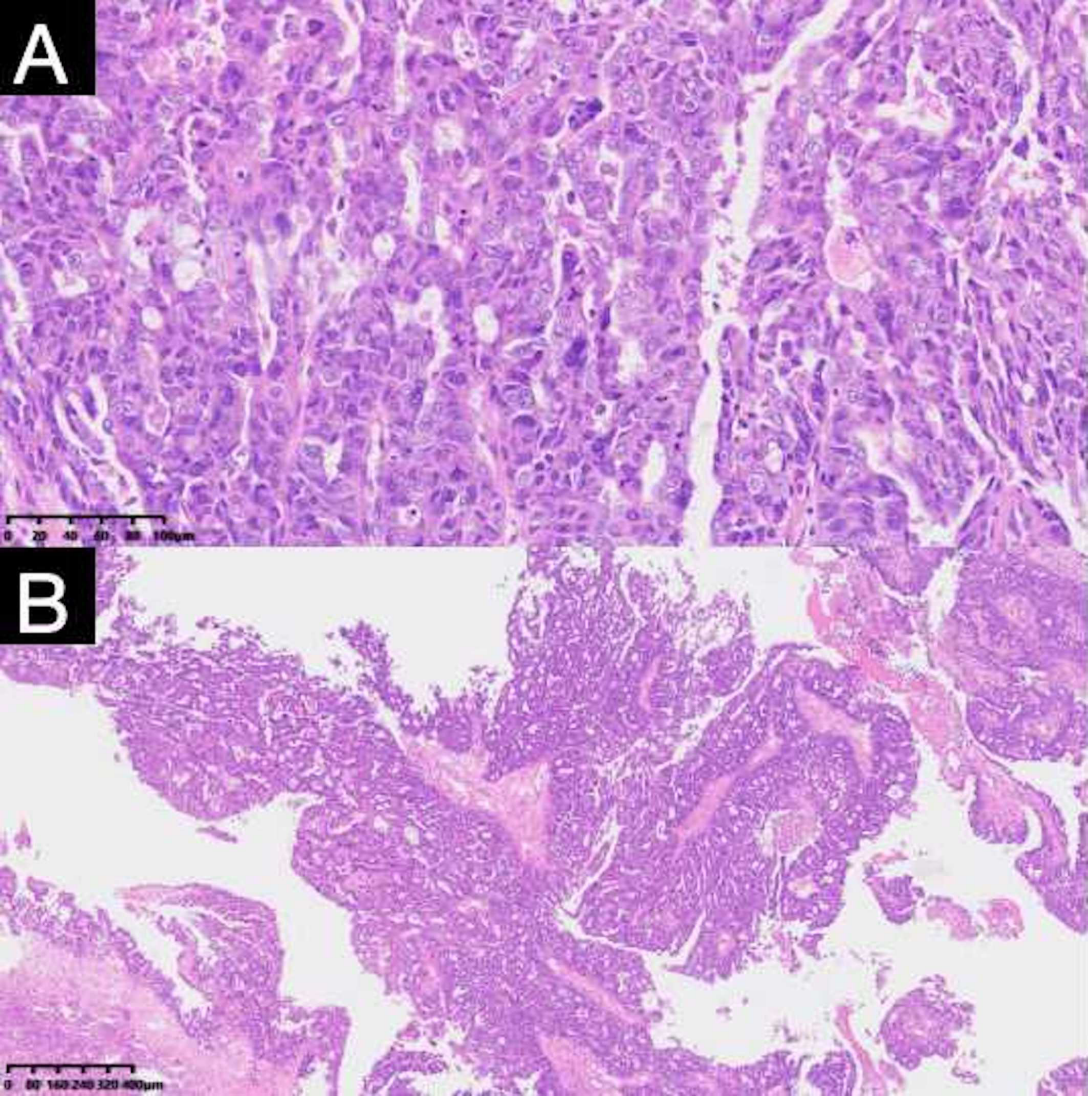
Pathologic findings of resected specimens from the patient. The postoperative pathological report showed high-grade serous carcinoma. **(A)** (H and E, ×100), **(B)** (H and E, ×40).

The patient generally maintains good physical health and has no other underlying diseases. The patient’s father passed away due to pancreatic cancer, and her mother passed away due to esophageal cancer. Genetic testing was negative for germline and somatic BRCA1/2 mutations. During the preoperative assessment, the patient reported experiencing persistent abdominal bloating for one month. Physical exam found hard masses in both adnexa. Carbohydrate antigen 125 (CA125) was 1725 U/mL. Ultrasound showed mixed masses in both adnexal regions with solid components suspected to be enlarged lymph nodes near the iliac vessels (**Figure 1**). Pelvic computed tomography (CT) revealed cystic and solid masses in both adnexa, suggesting malignancy. Imaging evaluation demonstrated metastatic involvement characterized by multiple enlarged lymph nodes (abdominal/retroperitoneal/pelvic) and peritoneal carcinomatosis with associated malignant ascites (**Figure 2**). For diagnostic and therapeutic purposes, the patient underwent laparoscopic exploration, which revealed a FIGO IIIC ovarian cancer with a FAGO TTI score of 8 (widespread peritoneal nodules 2 points, diffuse diaphragmatic infiltration 2 points, root of mesentery involvement 2 points, requiring bowel resection 2 points) in March 2022. According to the intraoperative findings, the patient was unable to undergo satisfactory tumor cell reduction surgery, so tissue biopsy was taken. The postoperative pathological report showed high-grade serous carcinoma (**Figure 3**). Subsequently, the patient received three cycles of neoadjuvant chemotherapy three cycles of neoadjuvant chemotherapy consisting of paclitaxel and carboplatin. Serial monitoring demonstrated a significant decline in CA-125 levels from 1725 U/mL at diagnosis to 31.15 U/mL post-treatment. Further imaging evaluation was conducted, abdominal ultrasound examination showed mixed masses in the bilateral adnexa and local peritoneal thickening in the pelvic cavity (**Figure 1**) and pelvic computed tomography (CT) imaging showed solid cystic masses in both adnexa, which were reduced in size compared to March 17, 2022. Apart from a significant reduction in the tumor bed, there were also fewer lymph nodes considered for metastasis compared to before (**Figure 2**). In June 2022, the patient ultimately underwent cytoreductive surgery, which included total hysterectomy, bilateral salpingectomy, oophorectomy, and small bowel mesenteric tumor debulking, achieving complete cytoreduction (R0). No enlarged lymph nodes were detected during the surgery. She then received 3 additional cycles of adjuvant chemotherapy, with normalization of CA-125. The last blood test showed a platelet count of 307 x 10^9^/L. After completion of adjuvant chemotherapy in August 2022, the patient was started on maintenance oral Niraparib (200 mg QD).

### 2.2 Development and intervention treatment measures for thrombocytopenia

In March 2023, approximately 6 months into Niraparib maintenance, the patient was found to have asymptomatic thrombocytopenia, with a platelet count of 89 x 10^9^/L. In light of this condition, Niraparib was held, and immediate initial treatment interventions were initiated. The patient was managed with subcutaneous interleukin-11 (150 μg daily). Despite ongoing treatment aimed at increasing the platelet count, the patient’s platelet level did not rise as anticipated; instead, it further declined to 66 x 10^9^/L in April 2023. Consequently, the patient was admitted for more comprehensive and specialized treatment.

### 2.3 Hospital admission and further management

On May 5, 2023, the patient was admitted due to severe thrombocytopenia with a platelet count of 7 x 10^9^/L. During the May 5–22 hospitalization, the platelet count fluctuated between 7–107 x 10^9^/L, requiring platelet transfusions, thrombopoietin (TPO) agonist therapy, and intermittent interleukin-11 treatment. After admission, we performed platelet antibody testing and a bone marrow aspiration examination for the patient, aiming to systematically identify other potential factors that may cause thrombocytopenia. Following rigorous analysis and evaluation, the bone marrow aspiration and biopsy showed mixed cytopenia and increased plasma cells, but no definitive diagnosis of immune thrombocytopenia. Both the patient’s platelet antibody test results and bone marrow aspiration examination were negative, further reinforcing our initial assessment that the patient’s thrombocytopenia may be related to specific treatment factors (such as adverse drug reactions) rather than other known causes.

**Figure 4:**
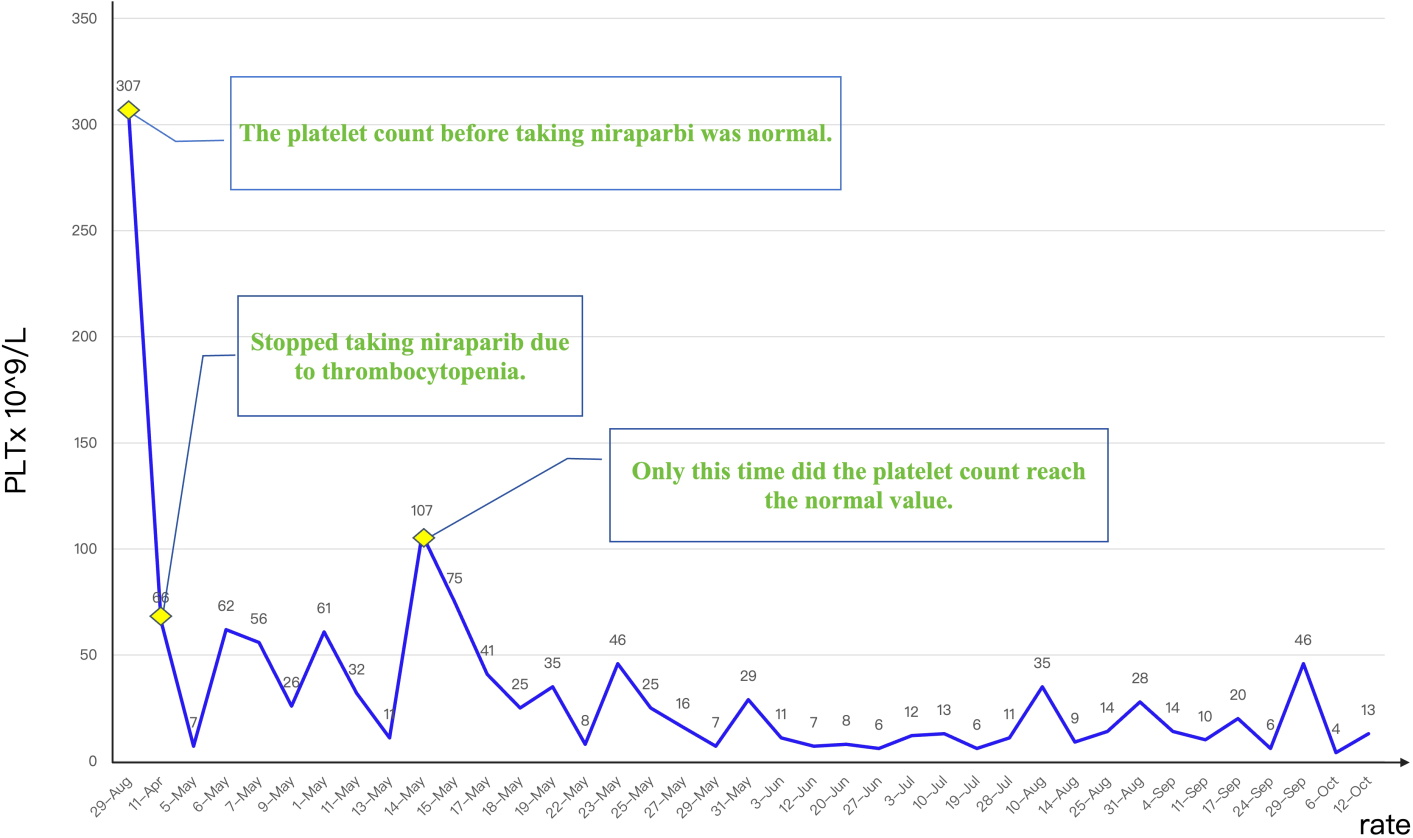
Platelet count trajectory before and following targeted therapy, demonstrating persistent thrombocytopenia despite ongoing treatment.

Given the ineffectiveness of pre-admission IL-11 therapy in stabilizing platelet levels, we initiated an aggressive treatment protocol comprising thrombopoietin (TPO) administration and prophylactic platelet transfusions to mitigate bleeding risk and promote hematopoietic recovery. Platelet transfusion is a direct method of supplementing platelets, which can rapidly increase the patient’s platelet count and prevent or treat bleeding symptoms caused by thrombocytopenia. **Figure 4** illustrates the longitudinal platelet count trajectory over seven months following thrombocytopenia onset. Despite multimodal therapeutic interventions, only transient normalization was achieved, suggesting refractory cytopenia with probable multifactorial pathogenesis.

During hospitalization, the treatment strategy was reevaluated in the context of refractory thrombocytopenia. Given the patient’s stable hematologic parameters before niraparib administration and the absence of alternative etiologies for thrombocytopenia, drug-induced myelosuppression was considered the primary etiology. Supportive measures, including platelet transfusions and hematopoietic agents, were prioritized to mitigate bleeding risk while maintaining oncologic surveillance. Although transient improvement was observed, the thrombocytopenia persisted. The patient subsequently underwent intensive hematologic monitoring to guide further management.

The patient was discharged, and outpatient management with oral platelet-stimulating agents and TPO agonists was continued. However, the patient’s condition deteriorated, with the development of liver metastases in October 2023 and subsequent death in December 2023.

## 3 Discussion

Niraparib, as a first-line PARP inhibitor in clinical practice, demonstrates a treatment discontinuation rate of 4%-12% primarily due to hematologic toxicities, which represent the most frequent grade 3–4 adverse events and major reasons for dose modification (12, 17, 18). The hematologic toxicity profile follows a characteristic temporal pattern, with thrombocytopenia typically emerging within the first 3 months of treatment (median onset: 4 weeks) and rarely occurring after 6 months (17). Mechanistically, this results from PARP1 inhibition in megakaryocyte lineage cells, which regulates hematopoietic stem cell differentiation and platelet production (19). The onset of platelet reduction can be insidious, presenting as asymptomatic thrombocytopenia or mucocutaneous bleeding, and in severe cases, internal organ hemorrhage or even fatal intracranial hemorrhage. Previous literature has also mentioned PARP inhibitor-induced thrombocytopenia, stating that the total incidence rate ranges from 16% to 61.3%, and the incidence of grade 3–4 thrombocytopenia (severe) is less than 1% to 33.8% (20). Specifically, for the patients receiving a fixed starting dose of Niraparib (200 mg once daily), the overall incidence rate is 61.3%, and the grade 3–4 incidence rate is 33.8%. In contrast, for those receiving an individualized starting dose (200 or 300 mg once daily), the overall incidence rate is 54%-54.8%, and the grade 3–4 incidence rate is 11.3%-21% (12, 18, 20, 21).

During PARP inhibitor therapy, rigorous monitoring of oral mucosa and gastrointestinal bleeding is essential to prevent life-threatening hemorrhage secondary (22). According to the NCCN Clinical Practice Guidelines for Hematopoietic Growth Factors, thrombocytopenia patients benefit from TPO receptor agonist romiplostim, which includes rhTPO, rhIL-11, and eltrombopag (23–26). When pharmacologic interventions prove ineffective, platelet transfusion remains the most rapid and effective method for correcting severe thrombocytopenia, significantly reducing the risk of major bleeding events and associated mortality. Prophylactic platelet transfusion is particularly indicated when platelet counts fall to ≤10×10^9^/L, especially in high-risk solid tumors (such as malignant melanoma, bladder cancer, gynecological tumors, and colorectal tumors) or hematological malignancies, where the threat of life-threatening hemorrhage is substantial (27). However, it should be noted that transfused allogeneic platelets have a short maintenance period of only 3–5 days and are rapidly consumed, so patients often require multiple transfusions. Platelet transfusion may also increase the risk of transfusion-transmitted infections and the development of platelet antibodies, leading to the ineffective transfusion or post-transfusion immune reactions.

This study systematically examines the clinical characteristics of niraparib-induced thrombocytopenia, explores management strategies, and discusses future research directions. For patients developing thrombocytopenia, primary management includes close platelet monitoring, appropriate dose adjustments of Niraparib, and temporary treatment interruption when necessary platelet recovery. While some studies have investigated prophylactic use of growth factors such as thrombopoietin receptor agonists, their efficacy remains uncertain. Platelet transfusion or other therapeutic interventions may be considered for refractory thrombocytopenia (14). However, in our report, available treatment proved ineffective against this persistent condition. As highlighted in previous analyses, the absence of predictive biomarkers for PARPi-related hematological adverse events (HAE) hinders the prevention and timely management (28). Consequently, accurately identifying this high-risk patient group has emerged as a pressing issue. While this report does not directly implicate age and weight as high-risk factors, it is generally acknowledged that elderly patients or those who are underweight or overweight may exhibit variations in drug metabolism and tolerance, necessitating their consideration as potential high-risk factors. Furthermore, a patient’s family history of tumors and long-term use of medications that impair bone marrow function can also elevate the risk of thrombocytopenia. Notably, although thrombocytopenia predominantly manifests within the first three months of treatment, as demonstrated in this case, some patients experience severe thrombocytopenia in later treatment stages. Therefore, when devising treatment timing and dosage adjustment strategies, these factors must be taken into account as crucial considerations for identifying high-risk populations.

For patients with high-risk factors, intensified clinical monitoring of blood counts should be commence at the initiation of treatment, with particular attention to changes following dosage adjustments. Additionally, patients undergoing PARP inhibitor therapy should be closely monitored for bleeding symptoms, such as mucosal and skin bleeding or visceral hemorrhage, which may serve as early warning signs of thrombocytopenia. For patients suspected of experiencing bone marrow suppression, further diagnostic procedures, including bone marrow aspiration and biopsy, should be conducted to comprehensively assess bone marrow function. Beyond clinical monitoring, enhancing patient education and follow-up is equally imperative. Comprehensive education should be provided to patients receiving Niraparib therapy, ensuring they are fully informed of the risks and warning signs associated with thrombocytopenia. Concurrently, efforts in follow-up and monitoring should be intensified to promptly detect and adequately address any emerging concerns.

Niraparib, as an effective PARP inhibitor, has demonstrated significant efficacy in cancer treatment. However, the thrombocytopenia induced by niraparib has emerged as a major clinical challenge. The underlying mechanism of this adverse effect are not fully elucidated, and its complexity may involve multiple interrelated factors. Bone marrow suppression, a common side effects of chemotherapy drugs, may exacerbate thrombocytopenia during Niraparib treatment. Additionally, the activation of autoimmune reactions cannot be overlooked, as it may trigger the production of antibodies against platelets, thereby accelerating platelet destruction. Further exploration of the pathogenesis of Niraparib-induced thrombocytopenia is essential. This will not only deepen our understanding of the mechanisms underlying this adverse effect but also provide a solid theoretical foundation for establishing predictive models. These models can help accurately identify high-risk patients and facilitate the development of personalized prevention and management strategies, thereby minimizing side effects while ensuring treatment effectiveness (29). On the other hand, as research on niraparib continues to advance, the development of new PARP inhibitors or combination therapies holds broad prospects. These new drugs or therapies may improve patient tolerance by reducing toxicity to normal cells or modulating immune responses while maintaining their original efficacy. This is expected to reduce the occurrence of side effects such as thrombocytopenia and further enhance the quality of life and treatment compliance of patients, offering safer and more effective treatment options for cancer patients.

## 4 Conclusion

This case report describes the first instance in real-world data of severe and persistent thrombocytopenia induced by Niraparib maintenance therapy in a patient with ovarian cancer. Despite systematic treatment, the patient still required weekly platelet transfusions to maintain adequate platelet counts. This case underscores the need for heightened clinical awareness and improved management strategies for refractory thrombocytopenia associated with Niraparib therapy.

## Data Availability

The original contributions presented in the study are included in the article/**Supplementary Material**. Further inquiries can be directed to the corresponding authors.
