# Correction: Severe thrombocytopenia induced by niraparib in ovarian cancer patients: a case report and literature review

**DOI:** 10.3389/fonc.2025.1661708

**Published:** 2025-07-24

**Authors:** Jiajun Li, Jiaxi Yang, Ying Wang

**Affiliations:** ^1^ Department of Pharmacology, Hebei Medical University, Shijiazhuang, China; ^2^ Department of Gynecology, The Fourth Hospital of Hebei Medical University, Shijiazhuang, China

**Keywords:** poly (ADP-ribose) polymerase inhibitor, niraparib, ovarian cancer, thrombocytopenia, case report

Author “Jiaxi Yang” was erroneously spelled as “Jiaqian Yang”.

In the published article, there was a mistake in the Author name. The Author name for the co-first author was misspelled as “Jiaqian Yang”. The correct spelling is “Jiaxi Yang’’

A correction has been made to the Keywords. The numbers after each keyword were removed as they did not carry significance.

The original version of this article has been updated.

