# Supplementary figures and images for: Severe thrombocytopenia induced by niraparib in ovarian cancer patients: a case report and literature review
Source: Front Oncol. 2025 Jul 4;15:1542646. doi: 10.3389/fonc.2025.1542646 (PMC12270851; doi:10.3389/fonc.2025.1542646)

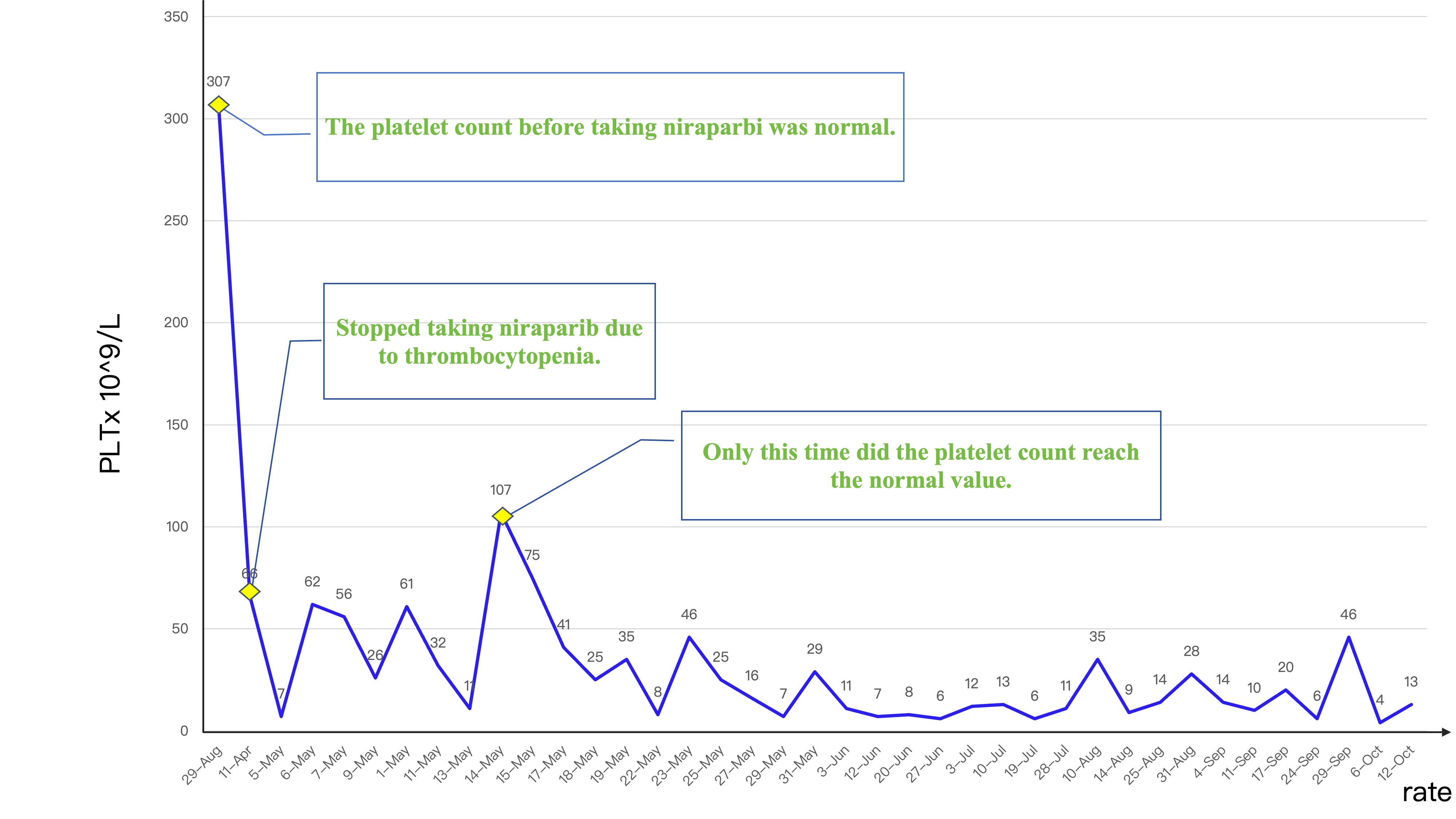

Supplement: Supplementary file 3 [file Image1.jpeg]
